# Enhanced 5-methylcytosine detection in single-molecule, real-time sequencing via Tet1 oxidation

**DOI:** 10.1186/1741-7007-11-4

**Published:** 2013-01-22

**Authors:** Tyson A Clark, Xingyu Lu, Khai Luong, Qing Dai, Matthew Boitano, Stephen W Turner, Chuan He, Jonas Korlach

**Affiliations:** 1 Pacific Biosciences, 1380 Willow Road, Menlo Park, CA 94025, USA; 2 Department of Chemistry and Institute for Biophysical Dynamics, 929 East 57th Street, The University of Chicago, Chicago, Illinois, 60637, USA

**Keywords:** Carboxylcytosine, DNA sequencing, epigenomics, methylation, methylcytosine, SMRT sequencing, Tet protein

## Abstract

**Background:**

DNA methylation serves as an important epigenetic mark in both eukaryotic and prokaryotic organisms. In eukaryotes, the most common epigenetic mark is 5-methylcytosine, whereas prokaryotes can have 6-methyladenine, 4-methylcytosine, or 5-methylcytosine. Single-molecule, real-time sequencing is capable of directly detecting all three types of modified bases. However, the kinetic signature of 5-methylcytosine is subtle, which presents a challenge for detection. We investigated whether conversion of 5-methylcytosine to 5-carboxylcytosine using the enzyme Tet1 would enhance the kinetic signature, thereby improving detection.

**Results:**

We characterized the kinetic signatures of various cytosine modifications, demonstrating that 5-carboxylcytosine has a larger impact on the local polymerase rate than 5-methylcytosine. Using Tet1-mediated conversion, we show improved detection of 5-methylcytosine using *in vitro *methylated templates and apply the method to the characterization of 5-methylcytosine sites in the genomes of *Escherichia coli *MG1655 and *Bacillus halodurans *C-125.

**Conclusions:**

We have developed a method for the enhancement of directly detecting 5-methylcytosine during single-molecule, real-time sequencing. Using Tet1 to convert 5-methylcytosine to 5-carboxylcytosine improves the detection rate of this important epigenetic marker, thereby complementing the set of readily detectable microbial base modifications, and enhancing the ability to interrogate eukaryotic epigenetic markers.

## Background

The DNA of most organisms is comprised of more than the four canonical bases (A, C, G and T). In mammals, for example, 5-methylcytosine (5mC) constitutes about 1% of all DNA bases and is found primarily in CpG dinucleotides. Methylation plays a critical role in the regulation of gene expression, genomic imprinting and the suppression of transposable elements [1]. Often referred to as the sixth base, 5-hydroxymethylcytosine (5hmC) is also found in many metazoan genomes [2]. 5hmC is converted from 5mC by the Ten-eleven translocation (Tet) family of proteins [3,4]. Recently, it was discovered that Tet proteins can also convert 5mC to 5-formylcytosine (5fC) [5] and 5-carboxylcytosine (5caC) [6,7]. In humans, there are three different Tet proteins (Tet1, Tet2, Tet3) that are all capable of this conversion [6,7]. It is currently thought that DNA demethylation may occur through this process of 5mC oxidation followed by base excision repair [6,8], and possibly decarboxylation [9].

Many of the genomes of bacteria and archea also contain modified DNA bases [10]. The three most common forms of methylation are 6-methyladenine (6mA), 4-methylcytosine (4mC) and 5mC. The primary function of methylation is DNA self-recognition via restriction-modification systems that protect the organism against invading DNA. However, there are methyltransferases (MTases), such as *dam*, that do not fall in restriction-modification systems and are important in chromosome stability, mismatch repair and replication [11]. There is some evidence that the presence of methylation can also impact gene expression [12]. Thus, detection and identification of methylated bases in both prokaryotes and eukaryotes is essential to the complete understanding of genome function.

The most common techniques for large-scale detection of DNA methylation rely on bisulfite treatment of the DNA prior to sequencing. Sodium bisulfite chemically deaminates cytosine residues to uracil, which are subsequently read out as thymine. Methylated cytosines are converted with much lower efficiency and thus remain cytosines. The presence of 5mC is inferred from comparing bisulfite-treated DNA sequences to an untreated reference. In standard bisulfite sequencing, 5mC cannot be distinguished from 5hmC [13]. The conversion of 5mC to 5caC through the activity of Tet1 [14] and 5hmC to 5fC through chemical conversion [15] followed by bisulfite sequencing runs has recently been exploited for the genome-wide sequencing of 5mC and 5hmC.

We have previously described a technique for the direct detection of modified DNA using single-molecule, real-time (SMRT^®^) sequencing [16,17]. SMRT sequencing involves the monitoring of a DNA polymerase as it makes a copy of a DNA molecule [18,19]. When the DNA polymerase encounters a modified base on the template strand, its rate of progression changes in a characteristic way relative to an unmodified template with the same sequence context [16,17]. The speed of the polymerase is monitored by determining the length of time between the fluorescent pulses that indicate nucleotide incorporation. The time between pulses is called the interpulse duration (IPD). The change in IPD between a modified and control template varies in magnitude and position depending on the nature of the base modification and the local sequence context. We refer to these reproducible changes in IPD as the kinetic signature for that modification.

Although many base modifications, such as 6mA, 4mC, 5hmC and 8-oxo-guanine, are readily detectable in SMRT sequencing [16,17,20,21], the kinetic signature of 5mC is more subtle, requiring high sequencing fold coverage to make out the small effect on polymerase speed. The methyl group is small, and unlike for the case of 6mA and 4mC, it is oriented towards the major groove and is not involved in base pairing - in fact the methyl group has to be readily accepted by DNA polymerases at this position as it is present on thymine, the other canonical pyrimidine base. We hypothesized that conversion of 5mC into a larger group may increase the magnitude of the kinetic signature during SMRT sequencing, thus enhancing the ability to detect 5mC. The Tet family of proteins carries out conversion of 5mC to several other modified forms of cytosine including 5hmC, 5fC and 5caC [6,7]. This strategy has been shown to be effective in the recently developed Tet-assisted bisulfite sequencing of 5hmC [14].

Here, we demonstrate that mouse Tet1 (mTet1) can be used to enhance direct detection of 5mC during SMRT sequencing. Using synthetic templates made from oligonucleotides containing 5mC, 5hmC, 5fC or 5caC modifications, we tested the kinetic signatures of each modification. We discovered that each of the moieties into which 5mC can be converted via Tet increased the magnitude of the kinetic signature, with 5caC having the largest effect. Next, we observed that oxidation of 5mC to 5caC on either synthetic templates or *in vitro *methylated DNA enhanced our ability to detect positions of 5mC. We then used our improved 5mC detection method for the genome-wide characterization of MTase activities in two different bacterial strains.

## Results

### SMRT sequencing shows varying kinetic signatures for different cytosine modifications

**Figure 1:**
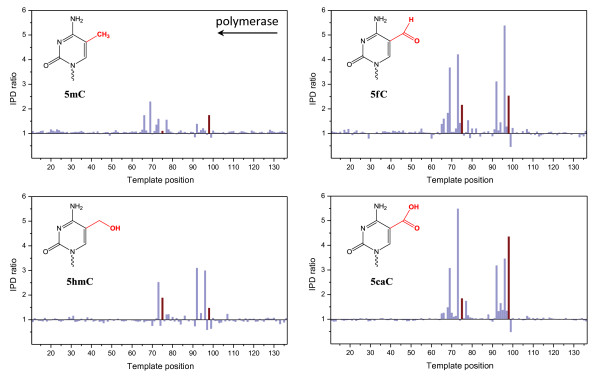
**Kinetic signals from SMRT sequencing for the four epigenetic markers of cytosine 5mC, 5hmC, 5fC and 5caC**. Synthetic oligonucleotides carrying two modified sites each (red bars) were subjected to SMRT sequencing and the polymerase kinetics compared by plotting the ratio of IPDs for each template position against a control template of identical sequence but lacking the modifications. The template is shown in the 5' to 3' direction from left to right, the polymerase movement is right to left across the template as indicated by the arrow. 5caC: 5-carboxylcytosine; 5fC: 5-formylcytosine; 5hmC: 5-hydroxymethylcytosine; 5mC: 5-methylcytosine; IPD: interpulse duration; SMRT: single-molecule: real-time.

To determine the kinetic signatures for the four naturally occurring forms of cytosine with a modification on the fifth carbon atom, we designed synthetic SMRTbell templates made from oligonucleotides with modified cytosines at specific template positions. Four modified synthetic SMRTbell templates were made, each containing two 5mC, 5hmC, 5fC or 5caC modifications. The polymerase dynamics of each was analyzed by SMRT sequencing and compared with a control template of the same sequence but lacking the modifications. The kinetic signatures for each cytosine modification type are shown in Figure 1 as ratios of the average IPD value at each template position of the modified template relative to the unmodified control. The positions of the modified bases are highlighted as red bars. As observed previously [17], the kinetic signature for 5mC is distinct from the background, but the magnitudes of the IPD ratios are small, translating to relatively high sequencing coverage for detection of the modified positions with high confidence. Furthermore, the kinetic signature is spread out over multiple positions on the DNA template [17], likely due to effects of base modifications on the polymerization rate extending across the entire footprint of the polymerase [22].

As the size of the chemical structure of the modification increases, the magnitude of the kinetic signature also increases. The IPD ratio peaks range from approximately two-fold for 5mC and approximately three-fold to higher than five-fold for 5fC and 5caC (Figure 1). For each modification type, an extended signature consisting of multiple IPD ratio peaks was observed, with the most prominent signals at positions 0, +2 and +6 relative to the polymerase movement, with 0 being the position of the modification in the template. In most instances investigated here, the +2 peak was the most pronounced. As previously observed [16,17], the kinetic signatures for a given modification varied slightly depending on the surrounding sequence context. These differences in the pattern and magnitudes of the kinetic signatures for each of the four different modifications are a parameter that can be used to discriminate between different modifications on the same DNA template, although they are not used in the current implementation of the software. To further explore the effects of local sequence context on the kinetic signatures of 5mC and 5caC, we used a synthetic SMRTbell template that contained a modified base in a 5'-CG-3' sequence context, surrounded by two random bases on each side. Additional file 1 shows a heat map of IPD ratios for the 256 possible sequence contexts at each position from -3 to +6 relative to the modified position in the template. As observed previously [17,20], the magnitude and position of the kinetic signals for both 5mC and 5caC are dependent upon the surrounding sequence context. The conversion of 5mC to 5caC enhances the magnitude of the IPD ratio at each position where ratios above 1.0 are observed for 5mC, that is, positions 0, +2, and +6, and brings out an additional detectable signal at the -2 position for some sequence contexts. Tet conversion enhances the kinetic signals relatively evenly across all sequence contexts, which is apparent from the good preservation of the overall sequence context profiles. We are currently investigating possible additional correlations that could exist between different base positions in a given context. This could aid in the development of more refined identification algorithms.

### Enhanced detection of 5mC following conversion to 5caC by mTet1

Because 5caC has the largest kinetic signature, conversion of 5mC to 5caC should significantly improve the ability to detect 5mC in SMRT sequencing. The Tet family of proteins has been shown to convert 5mC to 5caC in mammalian genomes [6,7]. This conversion can be over 97% for sequencing purposes and does not exhibit significant sequence context bias [14]. We tested the ability of Tet1-mediated oxidation of 5mC to 5caC to enhance direct detection on *in vitro *methylated DNA templates, described in detail in Methods. Briefly, we first generated an approximately 6-kb plasmid by inserting a lambda DNA fragment into the pCRBlunt vector and subjected it to whole genome amplification (WGA) to erase all modifications. We then generated an approximately 500 bp randomly sheared shotgun SMRTbell template library from the WGA material, followed by *in vitro *methylation using the HpaII MTase that modifies the internal cytosine in 5'-CCGG-3' sequence contexts. Considering both the forward and reverse DNA strands, the plasmid sequence contains 70 instances of the 5'-CCGG-3' sequence motif. Methylated positions within the SMRTbell templates were converted to 5caC by treatment with the Tet1 enzyme. *In vitro *methylated (5mC), Tet1 converted (5caC) and WGA control (no modification) libraries were then subjected to SMRT sequencing.

**Figure 2:**
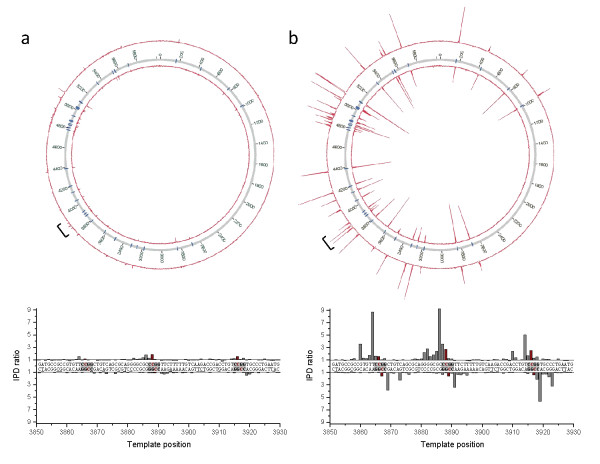
**Enhanced detection of 5mC through Tet1 oxidation using *in vitro *methylated plasmid samples**. The Circos plots show the plasmid-wide view of IPD ratios for **(a) **untreated and **(b) **mouse Tet1-treated samples, with the outer and inner circle denoting the forward and reverse DNA strands, respectively, and the blue tick marks denoting all positions of the targeted M.HpaII *in vitro *methylated sequence motif of 5'-CCGG-3' (methylated base underlined). The bracket denotes a section of the plasmid which is shown at base resolution in the bottom graphs, containing three instances of the methylated motif (grey boxes). The methylated positions are highlighted in red. IPD: interpulse duration.

Figure 2 shows the plasmid-wide view of IPD ratio data for the *in vitro *methylated (Figure 2a) and the mTet1-converted (Figure 2b) templates relative to the unmodified control. The IPD ratios for the 5mC-modified templates are visible as small excursions from the background (Figure 2a). Following mTet1-mediated oxidation to 5caC (Figure 2b), the kinetic signature was enhanced by an average of approximately 4.6-fold, making all 35 instances of the MTase recognition motif recognizable as large excursions in the IPD ratio. The primary IPD ratio peaks for the 5caC sample again fell at the +2 position relative to the modification, consistent with the results obtained from the synthetically derived samples. Similar results were obtained with synthetic SMRTbell templates that were made with oligonucleotides containing 5mC modifications and subjected to conversion by mTet1 (Additional file 2).

### Genome-wide analysis of 5mC DNA methyltransferase specificities in bacterial strains

Most bacterial and archeal genomes contain DNA MTases. Many of these MTases are paired with restriction endonucleases as part of a restriction-modification system that protects the organism from foreign DNA [23]. These MTases typically methylate a specific sequence context, which blocks the activity of the restriction enzyme that recognizes the same site. The three most common types of methylation found in bacteria and archea are 6mA, 4mC and 5mC. To test the ability of the mTet1-enhanced signal to detect 5mC in genomic DNA, we selected two bacterial strains that are known to express a 5mC MTase [24].

*Escherichia coli *K12 MG1655 is a well-studied, common laboratory strain that is known to express three different MTases. EcoKdam is a 6mA MTase that modifies the adenosine in a 5'-GATC-3' sequence context (methylated base underlined). EcoKI is a type I MTase that modifies the sequence context 5'-GCAC(N6)GTT-3' and reverse complement 5'-AAC(N6)GTGC-3'. The 5mC MTase is EcoKdcm that modifies the internal cytosine in a 5'-CCWGG-3', where W is either an A or a T. We made SMRTbell templates from randomly sheared *E. coli *K12 MG1655 genomic DNA, a portion of which was sequenced in its native form and another portion of which was subjected to the mTet1 treatment. Both samples were sequenced to approximately 150 × per-DNA strand fold coverage.

**Figure 3:**
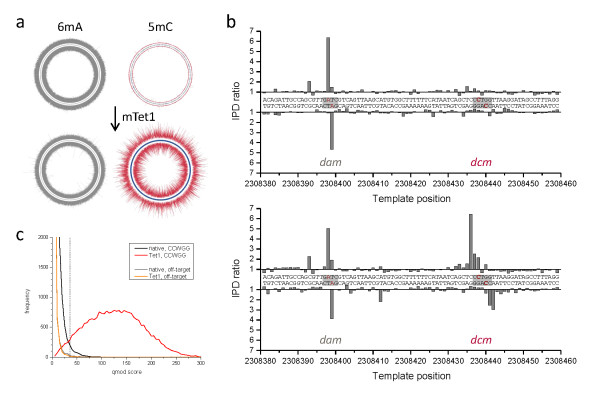
**Genome-wide 5mC methyltransferase specificity detection in *E. coli *K12 MG1655**. The Circos plots show the genome-wide view of IPD ratios for **(a) **untreated and **(b) **mTet1-treated samples, with the outer and inner circle denoting the forward and reverse DNA strands, respectively. The IPD ratios of the +2 position in 5'-CCWGG-3' sequence contexts are plotted in red, while IPD ratio data for all other contexts is plotted in grey. The graphs on the right show base-resolution IPD ratio views of a section of the genome containing one target site for adenine methylation by *dam *(5'-GATC-3') and one target site for cytosine methylation by *dcm *(5'-CCWGG-3'). **(c) **Kinetic score distributions before and after mTet1 conversion for all +2 positions of 5'-CCWGG-3' in the genome. An orthogonal off-target motif (5'-GGWCC-3') is also shown which was used to set a 1% false discovery rate threshold (dashed line, see Methods for details) for tabulation of detected methylated positions (Table 1). The blue tick marks in the Circos plots of (a) and (b) denote 5'-CCWGG-3' genomic positions detected as methylated using that threshold. 5mC: 5-methylcytosine; 6mA: 6-methyladenine; IPD: interpulse duration; mTet1: mouse Tet1.

We carried out an unbiased search for sequence motifs that were enriched in proximity to genomic positions with large excursions from the expected IPD values (see Methods for details). For the native sample we identified the expected 5'-GATC-3' and the 5'-GCAC(N6)GTT-3' and/or 5'-AAC(N6)GTGC-3' sequence motifs, but observed low signal levels for the 5'-CCWGG-3' motif. However, following the mTet1 conversion, we were able to identify the majority of 5'-CCWGG-3' motifs in the genome as modified. Figure 3 compares IPD ratio data over the entire *E. coli *genome before and after mTet1 treatment. As expected, IPD ratio data for sites methylated with m6A did not change between the native and Tet1-converted samples (panel a, grey lines). By contrast, IPD ratio data for the +2 position of the 5'-CCWGG-3' sites (panel a, red lines) were significantly increased in the mTet1-treated sample, thereby improving detection of *dcm*-mediated methylated positions, with IPD ratio magnitudes now similar to m6A signals. The distributions of IPD ratios for all methylated motifs are included in Additional file 3.

To estimate the degree of enhancement in 5mC detection by mTet1 treatment (Table 1), we selected the 99^th ^percentile kinetic score of an off-target motif (5'-GGWCC-3') as the threshold for calling a genomic position as methylated (Figure 3c). Any kinetic score that was greater than this value was considered modified. In the native sample, only 455 (1.9%) of all genomic 5'-CCWGG-3' positions were detected above this background value. Upon conversion of 5mC to 5caC in the mTet1-treated sample, 22,913 genomic 5'-CCWGG-3' positions (95.2%) were detected as methylated. The off-target site was unaffected by the mTet1 treatment, highlighting the specificity of the mTet1 conversion to methylated DNA sites. Additional file 4 shows the detection rate for all modified sequence motifs, including 6mA. This table also enumerates detection levels of additional off-target sequences that exhibit a consistently low percentage of sites above the detection threshold.

**Table 1:** Detection of 5mC in native versus mTet1-enhanced SMRT sequencing for the bacterial genomes

Sample		Methylation motif	Number in genome	Number detected	Number detected (%)	Number unassigned (%)^a^
*E. coli*	native	C^m^CWGG	24,079	455	1.9	0.4
MG1655	Tet1	C^m^CWGG	24,079	22,913	95.2	0.3
*B. halodurans*	native	GG^m^CC	15,207	660	4.3	0.6
C-125	Tet1	GG^m^CC	15,207	11,663	76.7	0.5

^a ^Unassigned is the percentage of genomic positions that have kinetic scores above the cutoff but are not in a methylated motif or a secondary peak.

**Figure 4:**
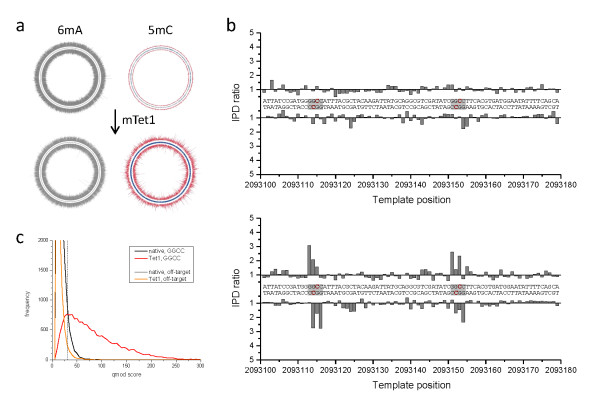
**Genome-wide 5mC methyltransferase specificity detection in *B. halodurans***. The Circos plots show the genome-wide view of IPD ratios for **(a) **untreated and **(b) **mTet1-treated samples, with the outer and inner circle denoting the forward and reverse DNA strands, respectively. The IPD ratios of the +2 position in 5'-GGCC-3' sequence contexts are plotted in red, while IPD ratio data for all other contexts is plotted in grey. The graphs on the right show base-resolution IPD ratio views of a section of the genome containing two target sites for cytosine methylation (5'-GGCC-3'). **(c) **Kinetic score distributions before and after Tet1 conversion for all +2 positions of 5'-GGCC-3' in the genome. An orthogonal off-target motif (5'-CCGG-3') is also shown which was used to set a 1% false discovery rate threshold (dashed line, see methods for details) for tabulation of detected methylated positions (Table 1). The blue tick marks in the Circos plots of (a) and (b) denote 5'-GGCC-3' genomic positions detected as methylated using that threshold. 5mC: 5-methylcytosine; 6mA: 6-methyladenine; IPD: interpulse duration; mTet1: mouse Tet1.

We performed the same procedure for *B. halodurans *C-125, a bacteriocin-producing soil bacterium. The *B. halodurans *genome is predicted to have three different MTases [24], including one MTase that has the hallmarks of a 5mC-modifying enzyme. However, unlike for the *E. coli *sample, the exact sequence motifs and positions of the modifications are not known. Through SMRT sequencing, we were able to identify two methylated sequence motifs: 5'-GCATC-3' or 5'-GATGC-3' and 5'-GGCC-3'. The first motif had high IPD ratio values on the A position on both forward and reverse strands, which is indicative of 6mA. This signal was present in both native and mTet1-treated samples (Figure 4). The 5'-GGCC-3' motif was considerably stronger in the mTet1-treated sample, with the strongest peak on the first G in the motif. Using the +2 pattern of the converted 5caC signature, the most likely modified base is the inner C in the 5'-GGCC-3' motif. We detected 4.3% of 5'-GGCC-3' motifs without mTet1-treatment, increasing to 76.7% following the enhancement of the 5mC signal by mTet1 conversion (Table 1). The distributions of IPD ratios for all methylated motifs in the *B. halodurans *genome are shown in Additional file 3 and the detection rate data are presented in Additional file 5.

## Discussion

In SMRT sequencing, modified bases in the DNA template are identified by the transient slowing of the DNA polymerase at and around the site of the modification. We previously demonstrated the detection of 5mC and 5hmC through such kinetic analysis [17]. Here, we extend the spectrum of detectable base modifications to the full complement of currently known modified forms of cytosine. Both 5fC and 5caC showed an increased interference with polymerase movement compared with 5mC, resulting in stronger kinetic signals in SMRT sequencing. In addition to the increased size of the modification, the higher polarity of the formyl and carboxyl group could also contribute to the increased signal levels.

In this work, we describe improving the direct SMRT sequencing of 5mC via mTet1-mediated oxidation to 5caC, thereby reducing the relatively high sequencing coverage required to detect the subtle signals imparted by 5mC with high confidence. mTet1 efficiently converted 5mC to 5caC in synthetic oligonucleotides, *in vitro *methylated plasmids, bacterial genomic DNA and mammalian genomic DNA [14], facilitating identification of microbial 5mC MTase specificities, thus complementing the other two common, readily detectable bacterial methylation marks of m6A and m4C described previously [16,17]. The protocol is rapid and specific to 5mC, allowing all three base modifications to be simultaneously detected in a single sequencing experiment. We anticipate that, for the sequencing of bacterial and archeal genomes, such comprehensive characterization of the methylome, in addition to *de novo *assembly of the genome [25,26], will improve our understanding of important microbiological phenomena, such as adaptation, pathogenicity and resistance evolution. It has been demonstrated through bulk biochemical and genetic studies that the dynamics of methylation in bacteria plays critical roles in basic cellular functions as well as directly affecting virulence [11,12,27].

The kinetic signatures of 4mC and 5caC are sufficiently different to allow for discrimination of the two types of cytosine modifications in bacteria. When sequencing through 4mC, the polymerase slows down only when incorporating the cognate nucleotide opposite the modification, with no significant secondary IPD ratio peaks [16]. By contrast, the primary IPD ratio peak for 5caC is located two bases after the modification (+2 position). The combination of observing the sequence identity and the specific kinetic signature make it possible to not only discover the presence of a base modification but also to determine the chemical identity of the type of modification. We are working on algorithmically harnessing this information contained in the kinetic signatures to expand the power of direct detection of modified bases unique to SMRT sequencing [28]. Algorithms that incorporate IPD data from multiple positions across the entire footprint of the polymerase may further enhance the ability to detect and discriminate between modification types. This multi-site analysis and a further understanding of the sequence context dependence of the 5caC kinetic signature should improve detection of 5caC, potentially reducing the sequencing coverage needed to detect converted 5mC positions even further.

In higher eukaryotes, the epigenome is much more complex as at least four different forms of cytosine can occur and dynamically interconvert at epigenetically regulated genomic positions. Emerging evidence suggests that the Tet proteins and the modified cytosines they generate are crucial for a growing list of biological processes, including zygotic epigenetic reprogramming, pluripotent stem cell differentiation, hematopoiesis and development of leukemia [2]. Thus, methods for comprehensive genome-wide mapping of all cytosine modifications will be critical for epigenomic studies. Several methods have been described recently for discriminating between 5mC and 5hmC using bisulfite sequencing in combination with chemical or enzymatic conversion [14,15]. Since for a given sequence context in SMRT sequencing, the kinetic signatures of 5mC, 5hmC, 5fC, and 5caC are different, there is the potential for direct identification of the various modifications on native DNA samples. We are working to expand the bioinformatics analysis algorithms towards discrimination of different epigenetic marks, taking into account the different signatures as a function of sequence context, as well as partial modification and mixtures of modification types. There are already several strategies for enhancing the kinetic signature of two cytosine modifications allowing for direct detection of 5mC and 5hmC in a single sample using SMRT sequencing. 5hmC positions can first be glucosylated [21], followed by Tet1-mediated oxidation of 5mC to 5caC. Glucosylated 5hmC will be protected from conversion and discrimination of the two forms can be made based on the differing kinetic signatures. We expect that these and further advances in the direct detection of modified bases during routine genome sequencing will become an important tool to further our understanding of genome and epigenome function.

## Methods

### Materials

Custom oligonucleotides containing modified bases were synthesized on-site or purchased from Trilink BioTechnologies (San Diego, CA, USA) and Integrated DNA Technologies (Coralville, IA, USA). All oligonucleotides contained 5' phosphate groups. The plasmid (pCRBlunt) was obtained from Life Technologies (Carlsbad, CA, USA). A list of the sequences can be found in Additional file 6.

Bacterial strains and/or genomic DNA from bacterial strains were purchased from the American Type Culture Collection (Manassas, VA, USA). The following strains were used in this study: *E. coli *K12 MG1655, and *B. halodurans *C-125 (JCM 9153).

### SMRTbell template preparation

Synthetic SMRTbell templates were made as previously described by ligating several synthetic oligonucleotides [20]. For plasmid and genomic DNA samples, an aliquot of approximately 25 ng of DNA was subjected to WGA using the REPLI-g Midi Kit (Qiagen, Valencia, CA, USA). WGA and native DNA was sheared to an average size of approximately 500 bp via adaptive focused acoustics (Covaris, Woburn, MA, USA). SMRTbell template sequencing libraries were prepared as previously described [16,29]. SMRTbell libraries made from whole-genome-amplified pCRBlunt-6K plasmid were *in vitro *methylated using the HpaII MTase (recognition sequence: 5'-C^5m^CGG-3'; New England BioLabs; Ipswich, MA, USA) as per the manufacturer's instructions. Complete methylation was assessed by modifying lambda DNA in parallel and subjecting to methylation-sensitive restriction using the HpaII restriction enzyme (New England BioLabs).

### Tet1 conversion

The 5mC modifications in SMRTbell template libraries were converted to 5caC using the 5mC mTet1 Oxidation Kit from Wisegene (Chicago, IL, USA) as per the manufacturer's instructions. Approximately 500 ng of SMRTbell templates were treated with the Tet1 enzyme at 37°C for 60 minutes followed by proteinase K treatment at 50°C for 60 minutes. Converted SMRTbell templates were purified using Micro Bio-Spin 30 Columns (BioRad, Hercules, CA, USA) with additional purification and concentration using MinElute PCR Purification Columns (Qiagen).

### Sequencing and data acquisition

SMRTbell templates were subjected to standard SMRT sequencing, as described [18,19]. Reads were processed and mapped to the respective reference sequences using the BLASR mapper [30] and Pacific Biosciences' SMRT Analysis pipeline [31] using the standard mapping protocol. IPDs were measured as previously described [17] and processed as described [16] for all pulses aligned to each position in the reference sequence.

For the bacterial methylome analysis [10], we used Pacific Biosciences' SMRTPortal analysis platform v. 1.3.1, which uses an *in silico *kinetic reference and a *t*-test based detection of modified base positions [32]. The following GenBank reference sequences were used: U00096.2 for *E. coli *K-12 MG1655 and BA000004.3 for *B. halodurans *C-125. MTase target sequence motifs were identified by selecting the top 1,000 kinetic hits and subjecting a ±20 base window around the detected base to MEME-ChIP [33], and compared to the predictions in REBASE [24]. To estimate the enhancement of detection of methylated 5mC positions (Table 1), we first selected an orthogonal off-target motif of similar sequence content and calculated the kinetic score representing the 99^th ^percentile of all genomic positions of that motif (5'-GGWCC-3' for *E. coli *(score threshold = 35.6); 5'-CCGG-3' for *B. halodurans *(30.4)). We then used this 1% false positive detection threshold for determining the number of genomic positions of the on-target methylation sites detected as methylated (Figures 3c and 4c; Table 1). IPD ratio plots were visualized using Circos [34].

## Additional data files

The following additional data are available with the online version of the paper. Additional data file 1 is a figure that demonstrates the sequence context dependence of the kinetic signatures for 5mC and 5caC. Additional data file 2 is a figure that shows IPD ratio data for synthetic SMRTbell templates before and after conversion of 5mC to 5caC. Additional file 3 is a figure with IPD ratio distributions for all methylated sequence motifs in *E. coli *and *B. halodurans*. Additional files 4 and 5 are tables that contain detection rate information for all methylated motifs in *E. coli *and *B. halodurans*, respectively. Additional data file 6 is a table of oligonucleotide sequences used in this study.

## Abbreviations

4mC: 4-methylcytosine; 5caC: 5-carboxylcytosine; 5fC: 5-formylcytosine; 5hmC: 5-hydroxymethylcytosine; 5mC: 5-methylcytosine; 6mA: 6-methyladenine; bp: base pair; IPD: interpulse duration; MTase: methyltransferase; mTet1: mouse Tet1; SMRT: single-molecule: real-time; Tet: Ten-eleven translocation; WGA: whole genome amplification.

## Competing interests

TC, KL, MB, ST, and JK are full-time employees at Pacific Biosciences, a company commercializing single-molecule, real-time nucleic acid sequencing technologies. The University of Chicago Office of Technology and Intellectual Property has filed patent application for the 5mC-oxidation strategy.

## Authors' contributions

TC, ST, CH, and JK conceived of the study. TC, XL, and QD carried out mTet1 conversions. TC and MB generated sequencing libraries and carried out the sequencing reactions. TC, KL, and JK carried out data analysis. TC and JK made figures and wrote the manuscript. All authors read and approved the final manuscript.

## Supplementary Material

Additional file 1 **Sequence context dependence of the kinetic signatures for 5mC and 5caC**. Top panel **(a) **is a schematic of the synthetic SMRTbell template with random bases surrounding 5mC or 5caC modifications in a CG sequence context. The modified position is indicated with pink text and an asterisk. The bottom panel **(b) **is a heat map of IPD ratio values of either 5mC or 5caC relative to an unmodified control sequence. IPD ratio values are shown for all possible sequence contexts of four random bases over ten positions on the DNA template (-3 to +6 relative to the modified base). Light grey boxes within the heatmap denote sequence contexts that did not have sufficient sequencing coverage. A minimum of 10 independent molecules of both modified and control templates were analyzed. Click here for file

Additional file 2 **Conversion of 5mC to 5caC in synthetic oligonucleotides**. Kinetic signals for synthetic oligonucleotides carrying two 5mC modified sites (red bars) are shown before (top) and after (bottom) mTet1-mediated oxidation to 5caC. IPD ratio data are plotted for each template position relative to a control template of identical sequence but lacking modifications. The template is shown in the 5' to 3' direction from left to right, the polymerase movement is right to left across the template as indicated by the arrow. Click here for file

Additional file 3 **IPD ratio distributions of all methylated motifs in *E.coli *MG1655 and *B.halodurans *C-125**. Each plots show the histograms of IPD ratio values for each methylated motif and an off-target non-methylated motif. The top plots are from native samples and the bottom show the same data after Tet1-mediated conversion of 5mC to 5caC. Click here for file

Additional file 4 **Table of detection rates for all methylated motifs in *E.coli *MG1655**. The number and percent detection is shown for all methylated sequence motifs in the genome. A detected genomic position is one that has a kinetic score that is greater than the cutoff value. Detection rates are also shown for common secondary IPD ratio peaks of 6mA (+5) and 5mC (+2, +6) and for off-target motifs with similar sequence content to the methylated motifs. Methylated bases are colored: 6mA (red), 5mC (blue). The interrogated base in the motif is underlined. Unassigned are genomic positions with kinetic scores above the cutoff which are not in a methylated motif or a secondary peak. Click here for file

Additional file 5 **Table of detection rates for all methylated motifs in *B.halodurans *C-125**. The number and percent detection is shown for all methylated sequence motifs in the genome. A detected genomic position is one that has a kinetic score that is greater than the cutoff value. Detection rates are also shown for common secondary IPD ratio peaks of 6mA (+5) and 5mC (+2, +6) and for off-target motifs with similar sequence content to the methylated motifs. Methylated bases are colored: 6mA (red), 5mC (blue). The interrogated base in the motif is underlined. Unassigned are genomic positions with kinetic scores above the cutoff which are not in a methylated motif or a secondary peak. Click here for file

Additional file 6 **Table of oligonucleotide sequences used in this study**. Click here for file
