# Supplementary material for: Enhanced 5-methylcytosine detection in single-molecule, real-time sequencing via Tet1 oxidation
Source: BMC Biol. 2013 Jan 22;11:4. doi: 10.1186/1741-7007-11-4 (PMC3598637; doi:10.1186/1741-7007-11-4)
Supplement: Additional file 1 — Sequence context dependence of the kinetic signatures for 5mC and 5caC. Top panel (a) is a schematic of the synthetic SMRTbell template with random bases surrounding 5mC or 5caC modifications in a CG sequence context. The modified position is indicated with pink text and an asterisk. The bottom panel (b) is a heat map of IPD ratio values of either 5mC or 5caC relative to an unmodified control sequence. IPD ratio values are shown for all possible sequence contexts of four random bases over ten positions on the DNA template (-3 to +6 relative to the modified base). Light grey boxes within the heatmap denote sequence contexts that did not have sufficient sequencing coverage. A minimum of 10 independent molecules of both modified and control templates were analyzed. [file 1741-7007-11-4-S1.PDF]

A

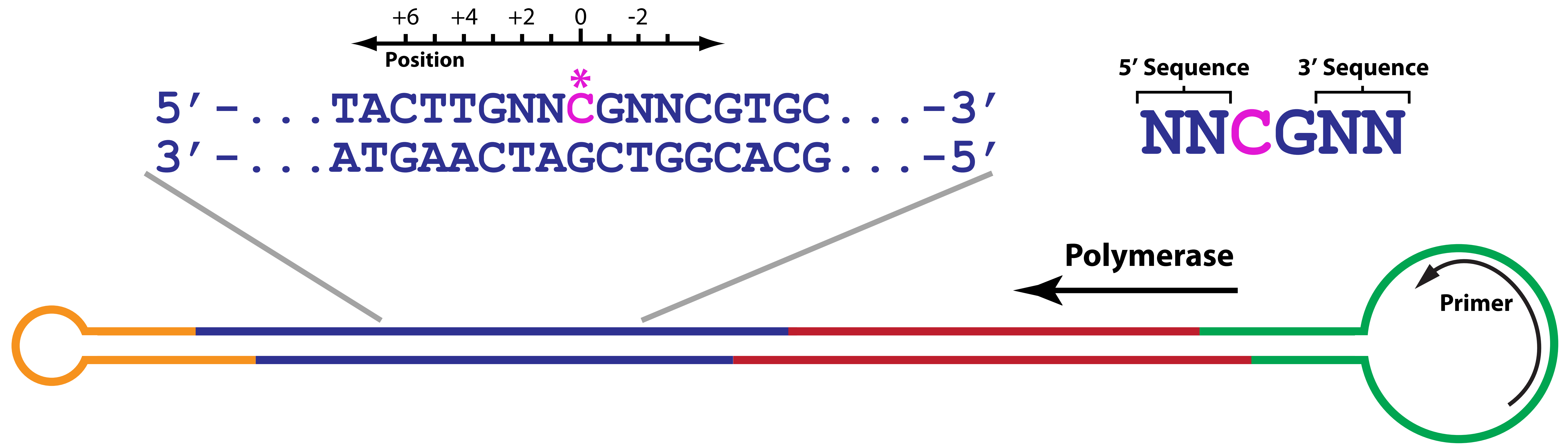
+6 +4 +2 0 -2
Position
5′–...TACTTGNNCGNNCGTGC...–3′
3′–...ATGAACTAGCTGGCACG...–5′
5′ Sequence
3′ Sequence
NNCGNN
Polymerase
Primer

B

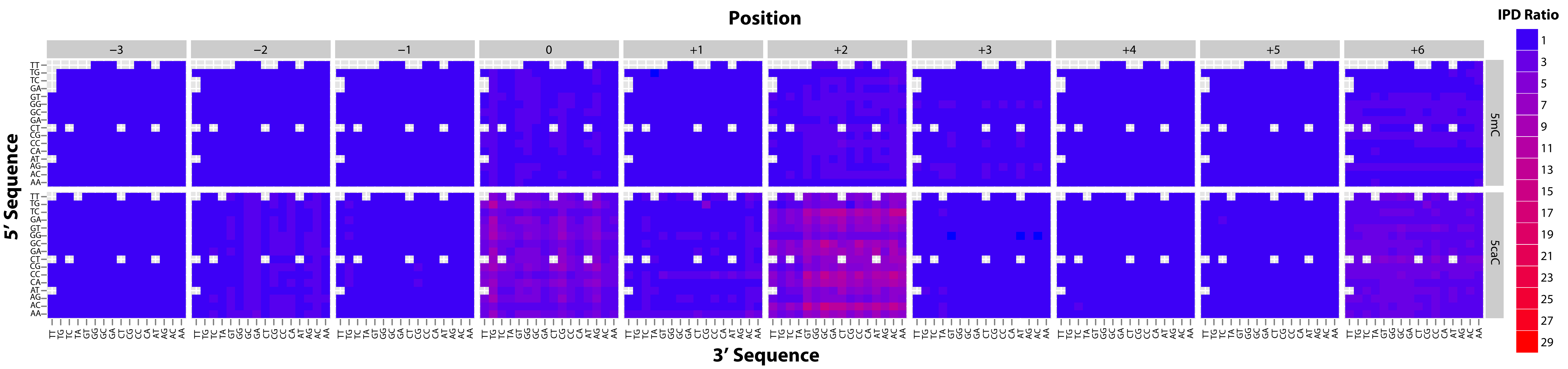
Position
−3 −2 −1 0 +1 +2 +3 +4 +5 +6
5mC
5caC
5′ Sequence
3′ Sequence
TT TG TC TA GT GG GC GA CT CG CC CA AT AG AC AA
IPD Ratio
1 3 5 7 9 11 13 15 17 19 21 23 25 27 29
