# Supplementary figures and images for: Enhanced 5-methylcytosine detection in single-molecule, real-time sequencing via Tet1 oxidation
Source: BMC Biol. 2013 Jan 22;11:4. doi: 10.1186/1741-7007-11-4 (PMC3598637; doi:10.1186/1741-7007-11-4)

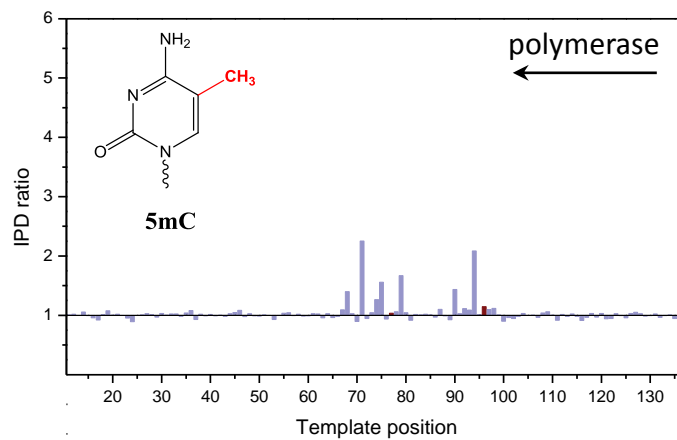

mTet1

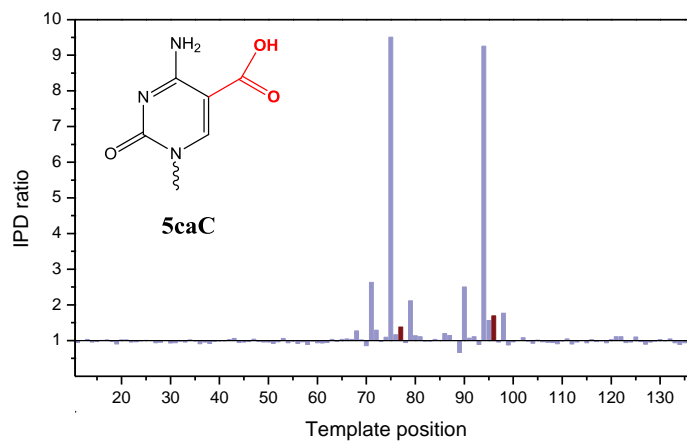

Supplement: Additional file 2 — Conversion of 5mC to 5caC in synthetic oligonucleotides. Kinetic signals for synthetic oligonucleotides carrying two 5mC modified sites (red bars) are shown before (top) and after (bottom) mTet1-mediated oxidation to 5caC. IPD ratio data are plotted for each template position relative to a control template of identical sequence but lacking modifications. The template is shown in the 5' to 3' direction from left to right, the polymerase movement is right to left across the template as indicated by the arrow. [file 1741-7007-11-4-S2.PDF]

# *E. coli* MG1655

Native

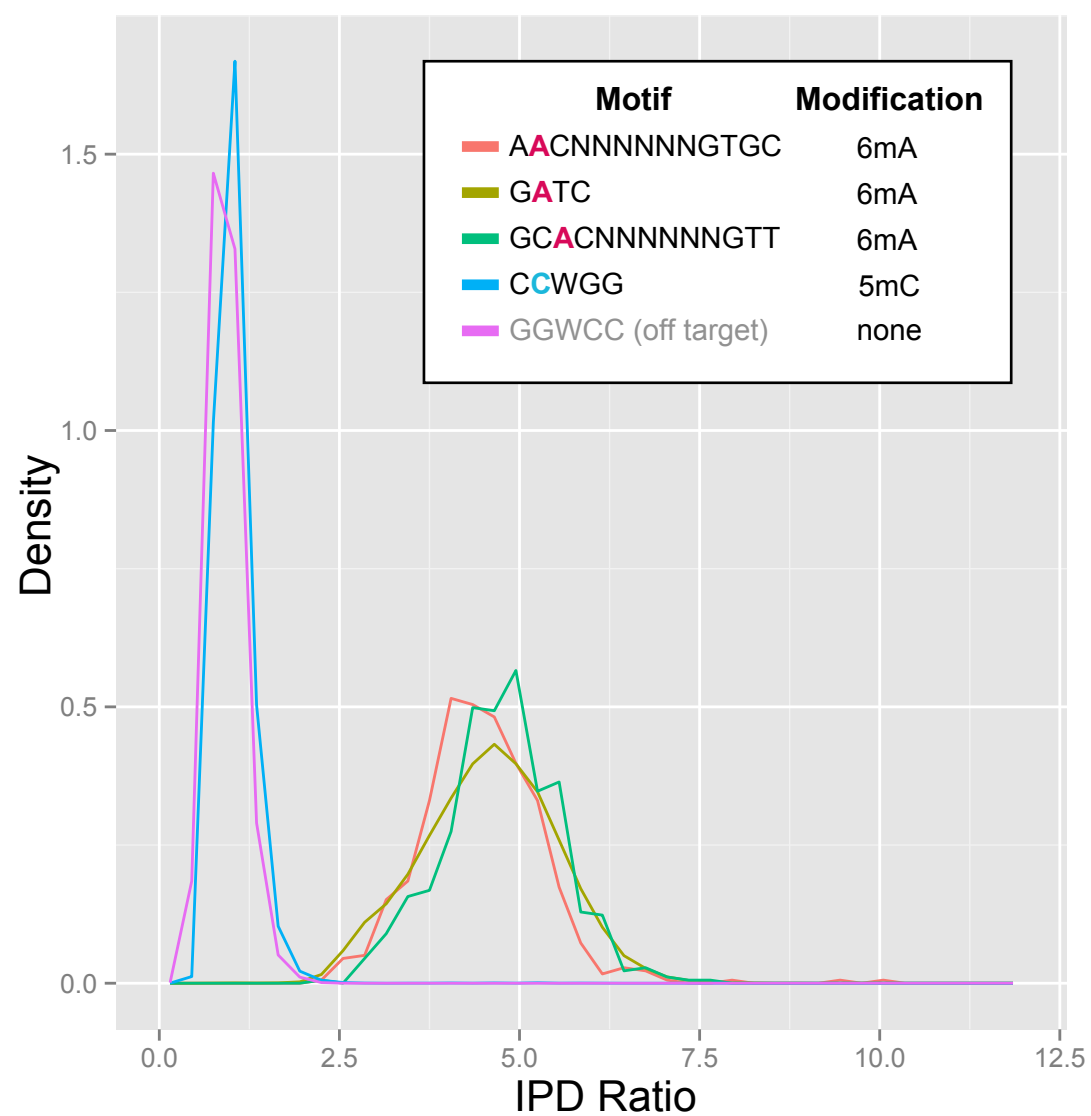

# *B. halodurans* C-125

Density

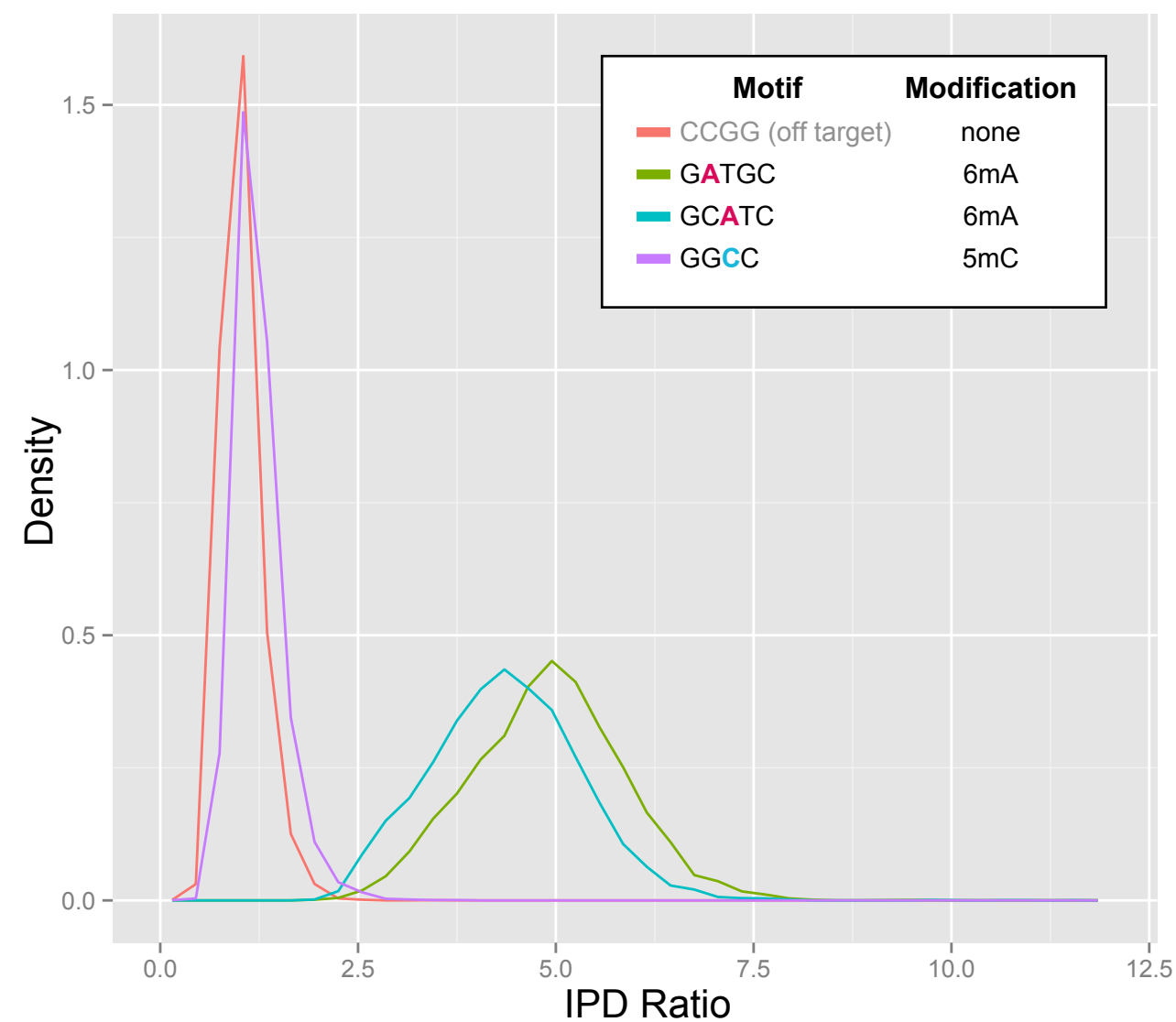

Tet1 Converted

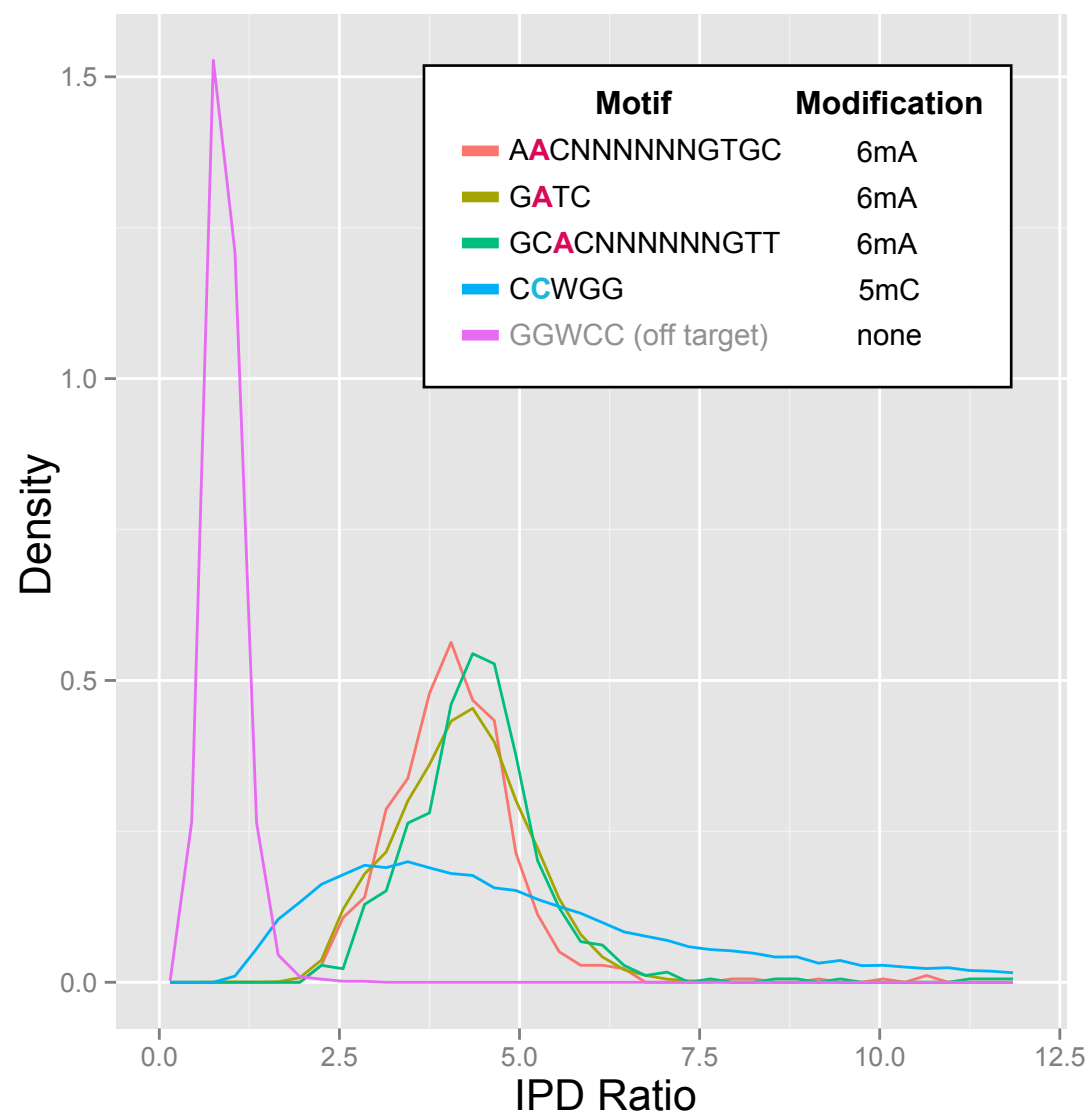

Density

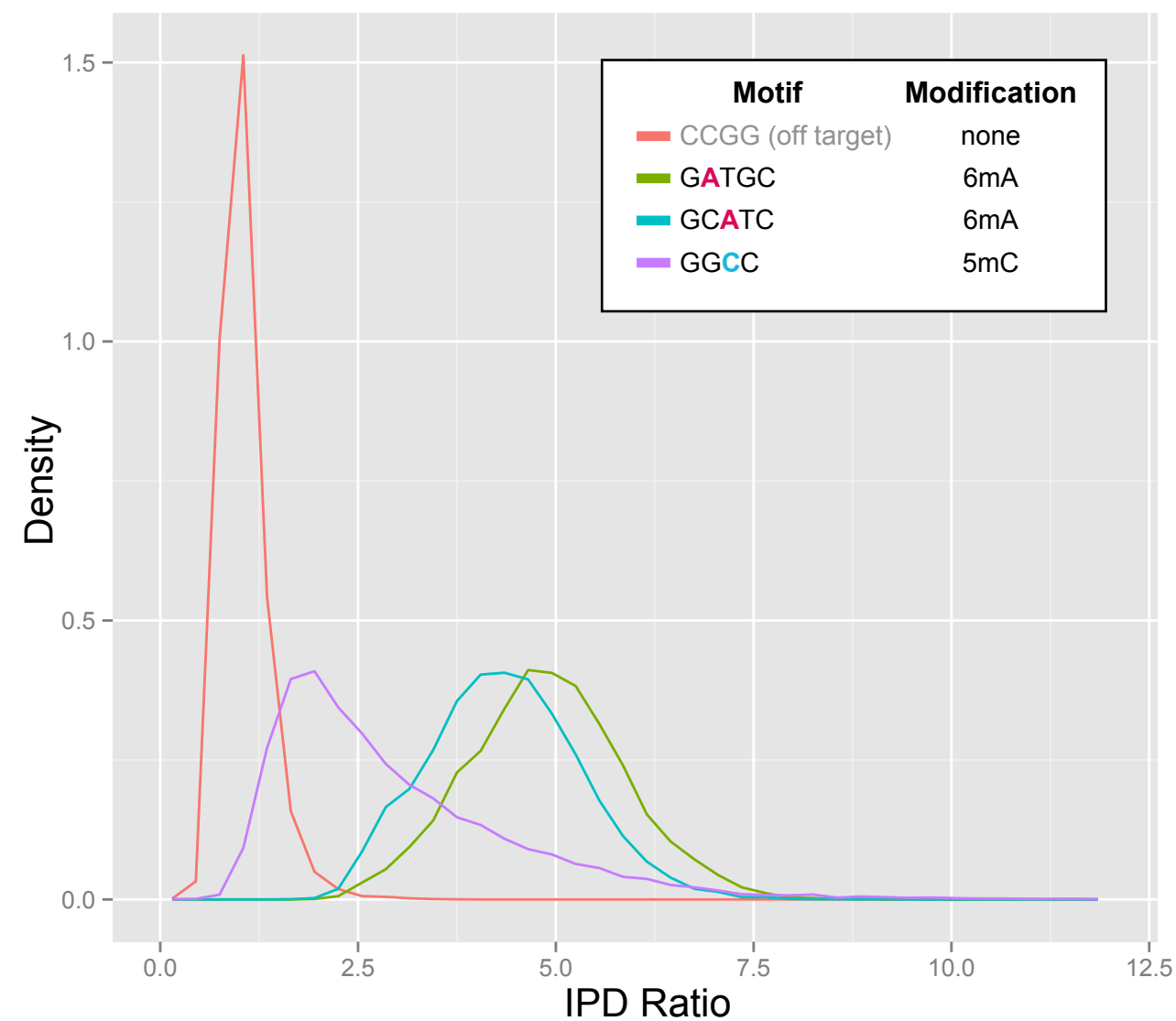

Supplement: Additional file 3 — IPD ratio distributions of all methylated motifs in E.coli MG1655 and B.halodurans C-125. Each plots show the histograms of IPD ratio values for each methylated motif and an off-target non-methylated motif. The top plots are from native samples and the bottom show the same data after Tet1-mediated conversion of 5mC to 5caC. [file 1741-7007-11-4-S3.PDF]
