# Supplementary material for: Enhanced 5-methylcytosine detection in single-molecule, real-time sequencing via Tet1 oxidation
Source: BMC Biol. 2013 Jan 22;11:4. doi: 10.1186/1741-7007-11-4 (PMC3598637; doi:10.1186/1741-7007-11-4)
Supplement: Additional file 4 — Table of detection rates for all methylated motifs in E.coli MG1655. The number and percent detection is shown for all methylated sequence motifs in the genome. A detected genomic position is one that has a kinetic score that is greater than the cutoff value. Detection rates are also shown for common secondary IPD ratio peaks of 6mA (+5) and 5mC (+2, +6) and for off-target motifs with similar sequence content to the methylated motifs. Methylated bases are colored: 6mA (red), 5mC (blue). The interrogated base in the motif is underlined. Unassigned are genomic positions with kinetic scores above the cutoff which are not in a methylated motif or a secondary peak. [file 1741-7007-11-4-S4.PDF]

Motif	Modification	# in Genome	Native # Detected	Native % Detected	Tet1 # Detected	Tet1 % Detected
5'-GATC-3'<br>3'-CTAG-5'	6mA	38235	38191	**99.9%**	38154	**99.8%**
5'-NNNNGATCNNNN-3'<br>3'-NNNNCTAGNNNN-5'	6mA (+5 peak)	38235	14885	**38.9%**	10011	**26.2%**
5'-GCACNNNNNNGTT-3'<br>3'-CGTGNNNNNNCAA-5'	6mA	595<br>595	594<br>595	**99.8%**<br>**100.0%**	594<br>594	**99.8%**<br>**99.8%**
5'-NNNGCACNNNNNNGTTNNNN-3'<br>3'-NNNCGTGNNNNNNCAANNNN-5'	6mA (+5 peak)	595<br>595	21<br>245	**3.5%**<br>**41.1%**	20<br>164	**3.4%**<br>**27.6%**
5'-CCWGG-3'<br>3'-GGWCC-5'	5mC	24079	715	**3.0%**	5648	**23.5%**
5'-NCCWGGN-3'<br>3'-NGGWCCN-5'	5mC (+2 peak)	24079	455	**1.9%**	22913	**95.2%**
5'-NNNNNCCWGGNNNNN-3'<br>3'-NNNNNGGWCCNNNNN-5'	5mC (+6 peak)	24079	717	**3.0%**	7192	**30.0%**
5'-CTAG-3'<br>3'-GATC-5'	Off Target	1767	13	**0.7%**	12	**0.7%**
5'-NNNNCTAGNNNN-3'<br>3'-NNNNGATCNNNN-5'	Off Target	1767	47	**2.7%**	27	**1.5%**
5'-CGTGNNNNNNCAA-3'<br>3'-GCACNNNNNNGTT-5'	Off Target	442<br>442	3<br>2	**0.7%**<br>**0.5%**	0<br>1	**0.0%**<br>**0.2%**
5'-NNNCGTGNNNNNNCAANNNN-3'<br>3'-NNNGCACNNNNNNGTTNNNN-5'	Off Target	442<br>442	0<br>3	**0.0%**<br>**0.7%**	0<br>3	**0.0%**<br>**0.7%**
5'-GGWCC-3'<br>3'-CCWGG-5'	Off Target	5736	18	**0.3%**	10	**0.2%**
5'-NGGWCCN-3'<br>3'-NCCWGGN-5'	Off Target	5736	60	**1.0%**	36	**0.6%**
5'-NNNNNGGWCCNNNNN-3'<br>3'-NNNNNCCWGGNNNNN-5'	Off Target	5736	16	**0.3%**	42	**0.7%**
Unassigned	Off Target	9128033	34831	**0.4%**	26833	**0.3%**

Additional File 4
