# Supplementary material for: Enhanced 5-methylcytosine detection in single-molecule, real-time sequencing via Tet1 oxidation
Source: BMC Biol. 2013 Jan 22;11:4. doi: 10.1186/1741-7007-11-4 (PMC3598637; doi:10.1186/1741-7007-11-4)
Supplement: Additional file 5 — Table of detection rates for all methylated motifs in B.halodurans C-125. The number and percent detection is shown for all methylated sequence motifs in the genome. A detected genomic position is one that has a kinetic score that is greater than the cutoff value. Detection rates are also shown for common secondary IPD ratio peaks of 6mA (+5) and 5mC (+2, +6) and for off-target motifs with similar sequence content to the methylated motifs. Methylated bases are colored: 6mA (red), 5mC (blue). The interrogated base in the motif is underlined. Unassigned are genomic positions with kinetic scores above the cutoff which are not in a methylated motif or a secondary peak. [file 1741-7007-11-4-S5.PDF]

Motif	Modification	# in Genome	Native # Detected	Native % Detected	Tet1 # Detected	Tet1 % Detected
5'-GATGC-3'<br>3'-CTACG-5'	6mA	7830<br>7830	7794<br>7785	**99.5%**<br>**99.4%**	7780<br>7779	**99.4%**<br>**99.3%**
5'-NNNNGATGCNNN-3'<br>3'-NNNNCTACGNNN-5'	6mA (+5 peak)	7830<br>7830	2934<br>159	**37.5%**<br>**2.0%**	2486<br>132	**31.7%**<br>**1.7%**
5'-GGCC-3'<br>3'-CCGG-5'	5mC	15207	1227	**8.1%**	7264	**47.8%**
5'-GGCC-3'<br>3'-CCGG-5'	5mC (+2 peak)	15207	660	**4.3%**	11663	**76.7%**
5'-NNNNGGCCNNNN-3'<br>3'-NNNNCCGGNNNN-5'	5mC (+6 peak)	15207	436	**2.9%**	1077	**7.10%**
5'-CTACG-3'<br>3'-GATGC-5'	Off Target	3662<br>3662	29<br>25	**0.8%**<br>**0.7%**	16<br>21	**0.4%**<br>**0.6%**
5'-NNNNCTACGNNN-3'<br>3'-NNNNGATGCNNN-5'	Off Target	3662<br>3662	103<br>29	**2.8%**<br>**0.8%**	58<br>61	**1.6%**<br>**1.7%**
5'-CCGG-3'<br>3'-GGCC-5'	Off Target	12671	139	**1.1%**	524	**4.1%**
5'-CCGG-3'<br>3'-GGCC-5'	Off Target	12671	124	**1.0%**	245	**1.9%**
5'-NNNNCCGGNNNN-3'<br>3'-NNNNGGCCNNNN-5'	Off Target	12671	64	**0.5%**	95	**0.7%**
Unassigned	Off Target	8327763	51955	**0.6%**	38406	**0.5%**
