# Supplementary material for: Enhanced 5-methylcytosine detection in single-molecule, real-time sequencing via Tet1 oxidation
Source: BMC Biol. 2013 Jan 22;11:4. doi: 10.1186/1741-7007-11-4 (PMC3598637; doi:10.1186/1741-7007-11-4)
Supplement: Additional file 6 — Table of oligonucleotide sequences used in this study. [file 1741-7007-11-4-S6.PDF]

Name	Sequence	Modification
Top-Left(TL)_control	cccgACGCATGATCTGTACTTGATCGACCGTGcaac	
Bottom-Left	CACGGTCGATCAAGTACAGATCATGCGT	
Top-Right	GCATGATCTGTACTTGATCGACCGTGCA	
Bottom-Right	gaagTGCACGGTCGATCAAGTACAGATCATGCgttg	
Hairpin-Left	cgggCTCGGAACgaaaGTTCCGAG	
Hairpin-Right	cttcTCTCTCTCttt tcctcctcctccgtt gttgttgttGAGAGAGA	
TL_5mC	cccgAxGCATGATCTGTACTTGATCGACxGTGcaac	x = 5-Methylcytosine
TL_5hmC	cccgAxGCATGATCTGTACTTGATCGACxGTGcaac	x = 5-Hydroxymethylcytosine
TL_5fC	cccgAxGCATGATCTGTACTTGATCGACxGTGcaac	x = 5-Formylcytosine
TL_5caC	cccgAxGCATGATCTGTACTTGATCGACxGTGcaac	x = 5-Carboxylcytosine
TL_Random_Control	cccgTAGAACGTGTACTTGNNCGNNCGTGCAAGCTAGTcaac	
TL_Random_5mC	cccgTAGAACGTGTACTTGNNxGNNCGTGCAAGCTAGTcaac	x = 5-Methylcytosine
TL_Random_5caC	cccgTAGAACGTGTACTTGNNxGNNCGTGCAAGCTAGTcaac	x = 5-Carboxylcytosine
BL_Random	ACTAGCTTGCACGGTCGATCAAGTACACGTTCTA	

Additional File 6
